# A Large Sample Survey of Tibetan People on the Qinghai–Tibet Plateau: Current Situation of Depression and Risk Factors

**DOI:** 10.3390/ijerph17010289

**Published:** 2019-12-31

**Authors:** Jiazhou Wang, Yueyue Zhou, Yiming Liang, Zhengkui Liu

**Affiliations:** 1 CAS Key Laboratory of Mental Health, Institute of Psychology, Beijing 100101, China; wangjz@psych.ac.cn (J.W.); zhouyueyue@psych.ac.cn (Y.Z.);; 2 Department of Psychology, University of Chinese Academy of Sciences, Beijing 100049, China

**Keywords:** Qinghai–Tibet plateau, Tibetan people, mental health, depression, high altitude

## Abstract

Background: A depressive state is a negative emotional state characterized by abnormal dejection and unpleasant mood. Long-term depressive symptoms can result in psychological disorders such as depression. However, little is known about the depression status and risk factors of the Tibetan people on the Qinghai–Tibet Plateau. Objective: This study explores the depression status of the Tibetan people to better promote ethnic minorities’ physical and mental health. Participants and Setting: The Center for Epidemiologic Studies Depression Scale (CES-D) was administered to 24,141 Tibetan people from Yushu Prefecture; the average age was 34.33 years (SD = 9.18, range = 18–94 years). Materials and Methods: Participants completed questionnaires collecting demographic information and evaluating symptoms of depression. Results: The depression prevalence was higher at high altitudes, and there may be a significant positive correlation between depression rates and altitude. Significant differences were found for each demographic variable. Participants with depressive symptoms (scores ≥8) accounted for 52.3% of the total sample, and participants with depression (scores ≥14) accounted for 28.6%. The binary logistic regression results showed that alcohol drinkers, unmarried participants, participants with high self-assessed socioeconomic status, participants with a high income level, and those with a middle-school education were more likely to be depressed. Conclusions: The results provide the first evidence that the prevalence of depression in Tibetans of the Qinghai–Tibet Plateau is higher than that in the general Chinese population and that reported in Western studies, a finding that may be related to cultural differences and chronic hypoxia caused by the high altitude. This paper offers insight into the mental health status of people living in plateau areas and provides a basis for formulating pertinent mental health policy.

## 1. Introduction

A depressive state is a negative emotional state characterized by abnormal dejection and unpleasant mood; as such, it is a common experience of everyday life. Long-term depressive symptoms can lead to depression and other psychological disorders. As a syndrome or disorder, depression can be caused by a variety of causes and is characterized by a marked and persistent state of low mood or loss of interest or pleasure accompanied by a range of physical symptoms [1]. Although depression is a chronic disease, it can cause great harm and is associated with a high disability rate. At its worst, depression can lead to suicide [2]. According to the World Health Organization (WHO), depression is the single largest cause of nonfatal health loss worldwide (i.e., all years lived with disability) and the major contributor to suicide deaths [3]. Given the extreme adverse effects of depression on society, families, and individuals, numerous researchers have examined the risk factors for depression to develop effective prevention and intervention strategies [4,5,6].

Regarding the risk factors for depression, genetic and psychosocial factors have received the most attention. The role of genes as risk factors in this field is often discussed because of the complicated etiology and pathological mechanism of depression [7,8]. Monoaminergic and glutamatergic systems have been shown to inhibit depression [9,10,11]. Meanwhile, numerous psychosocial risk factors associated with depression have been identified. For example, lower life satisfaction and less social support were found to be risk factors for depression [12,13,14,15]. In addition, some demographic factors, such as occupation, also affect depression levels [16]. Although previous studies have provided outstanding insights into the etiology of depression, natural environmental factors have largely been neglected, and the geographical environments in which depressed people live need to be further explored.

Previous studies have shown that depression may be more severe at higher altitudes. The possible reasons for this phenomenon are as follows: the relatively weak community support caused by low population density, lack of access to mental health resources [17,18], high-altitude hypoxia [19,20], and insomnia [21]. For example, based on the biogenic amine theories of depression, the effects of hypoxia on serotonin and catecholamines suggest that hypoxia can contribute to depression [22]. Even when demographic factors such as age, gender, race, and income are controlled for, the occurrence of depression is significantly correlated with climatic pressure, especially with altitude [23]. People living in high-altitude areas are more likely to suffer from hypoxemia because of their unique living environment [24,25]. The combined effects of adverse natural conditions, low atmospheric pressure, melatonin, pharmacokinetics, and metabolism on mood and mental state may increase the severity of depression among those living in high-altitude environments [26,27,28].

The total number of people living in plateau areas worldwide is approximately 400 million, but most of them live in valleys at lower altitudes [29]. Yushu Tibetan Autonomous Prefecture in western China is located in the eastern hinterland of the Qinghai–Tibet Plateau and has an average altitude of 4493.4 m [30]. It is one of the poorest areas in China. The Tibetan people here belong to one of the two largest human plateau-dwelling groups in the world; they are also the oldest plateau group and live at the highest altitude [31]. Furthermore, the study of the human plateau-dwelling population itself is of immense significance. The group’s narrow geographical range and historical isolation in terms of marriage customs has caused extreme genetic isolation, providing ideal conditions for the study of the plateau population. However, due to the rugged geographical environment, lack of basic resources and facilities, and low population density, it is difficult to obtain health status information on ethnic minorities in remote areas of western China [32]. Compared with China as a whole, this area’s socioeconomic poverty and geographical location have translated into poor mental health status and poor medical aid [33]. For example, previous studies have shown that the lack of independent and objective data on Tibetans has been an obstacle to improving their physical and mental health [34]. Therefore, it is necessary to pay attention to and study their mental health [35,36].

In summary, the current situation of depression and risk factors among Tibetans on the Qinghai–Tibet Plateau is worth discussing. This research aimed to investigate depression and risk factors in Tibetans in Yushu Prefecture, Qinghai Province, and to understand the prevalence of depression and the influence of demographic factors on differences in depression symptoms to provide a scientific basis for future research.

## 2. Materials and Methods

### 2.1. Participants

The study was based on data from a large-scale joint survey conducted by the Institute of Psychology of the Chinese Academy of Sciences and the National Health and Family Planning Commission’s Institute of Science and Technology to understand the physical and mental health of adult Tibetans in China. In this study, a total of 30,000 questionnaires were issued; of these, 28,288 questionnaires were collected and 4147 invalid questionnaires were excluded (because of missing data or failure to answer the questions seriously). The final total number of valid questionnaires was 24,141, with a male/female ratio of 1:1.42 (9965 males and 14,176 females), and the effective rate was 85.3%. The average age of the respondents was 34.33 years (SD = 9.18, range = 18–94 years). The inclusion criteria were as follows: (1) minimum age of 18 years at the time the questionnaire was completed, (2) born and live in Yushu Prefecture for a long time, and (3) ethnic Tibetan. For exclusion criteria, those who had a history of mental illness were excluded.

All of the participants followed Tibetan Buddhism and spoke minority languages, but most of them understood Chinese well. For the very few participants who were not proficient in Chinese, our experimenter interpreted and translated the questionnaire. The demographic characteristics of the sample were as follows: 74.6% of the sample was under the age of 40 years, and only 2% of the sample was aged 60 years or older; 65.4% of the participants rated their socioeconomic status as medium. Only 0.2% of the participants were from families with an annual income of more than 200,000 yuan. Those with a middle school education or below were the majority, accounting for 92.9%; 89.2% of participants were married to their first spouse, and 94.7% were satisfied with their marital status. The overall rate of alcohol consumption was just 2.2%.

### 2.2. Procedure

This cross-sectional study was conducted from September to November 2016 in Yushu Prefecture and used a combined cluster sampling and convenience sampling design to select participants. In Yushu Prefecture, all adult residents in six county-level administrative regions were included in the study’s sample, and the Mother and Children Health Hospital in each county was responsible for issuing a notice to the township hospitals within its jurisdiction for participants to complete questionnaires at their township hospitals. However, in a small number of townships, some of the participants’ home addresses were hundreds of kilometers away from the test site, and there was no traffic between the two locations, making it unfeasible for all individuals in the cluster to be included in the sample.

All the research assistants received standardized and strict training before the investigation. The training included the introduction of the study, the rules and skills of reading informed consent and providing guidance, and potential difficulties that might arise during the testing process and how to address them. The research assistants dictated the contents of the questionnaire in Chinese to the participants, and the participants provided oral answers, which were then recorded by the research assistants. Several participants who were not proficient in Chinese completed the questionnaire with the help of research assistants who were proficient in both Chinese and Tibetan. The participants were informed that their responses would be completely confidential. Participation in the survey was completely voluntary. Before the implementation of the survey, the participants signed the informed consent form, and the questionnaire was collected on the spot. Finally, all the participants were entitled to a free medical examination as compensation for their participation. All the study procedures were approved by the research ethics review board of the Institute of Psychology, Chinese Academy of Sciences (ethical approval number: H16014).

### 2.3. Measures

Depression. This study used the 10-item Chinese short version of the Center for Epidemiologic Studies Depression Scale (CES-D) to assess depression symptoms. The CES-D was developed by Radloff [37], who was on the staff of the United States National Institutes of Health (NIH) in 1977. Subsequent researchers adapted items 1, 5, 6, 7, 8, 10, 11, 12, 14, and 20 to form the 10-item simplified version [38]. Chinese researchers translated and revised the simplified version to create the Chinese version and establish national urban norms for China [39]. This scale was used to investigate the frequency with which the participants had experienced depression symptoms in the last week. It is a 10-item scale with a three-factor structure. The factor structures are negative mood (items 2, 4, 7, and 10), positive emotions (items 5 and 8), and somatic symptoms (items 1, 3, 6, and 9).

The CES-D is widely used worldwide to screen for depression in the general population. Countless Chinese studies have also used this scale in different populations, and the reliability and validity of the Chinese version have been evaluated [40,41]. Because the participants in this study were Tibetans, whose level of education is generally low, the use of an oral questionnaire was necessary; thus, the use of a simple scale could significantly reduce response stress for the participants [42]. The scale items are rated from 0 (almost never or not at all) to 3 (most of the time), and the higher the score is, the higher the depression level. Radloff, the original author of the 20-item CES-D, initially recommended using 16 points as the cut-off for possible depression problems (depression symptoms, present in 20% of the population) and later suggested using 28 points as the cut-off for depression status (high risk of depression, present in 5% of the population) [37,43].

Chinese researchers hold that the cut-off recommended by Radloff reflects research results from the 1970s in the United States. Due to the passage of time and cultural differences between the East and the West, the original demarcation should be adjusted for research with Chinese participants [39]. Therefore, referring to the relevant studies of the original author and Chinese researchers, this study defined scores greater than or equal to 8 as indicative of depression symptoms and scores greater than or equal to 14 as indicative of depression [44,45,46,47]. In this study, the scale had high reliability and validity, and its Cronbach alpha coefficient was 0.923.

Demographic variables. Considering the possible demographic differences among Tibetan adults, we also measured several important demographic variables: gender, age, alcohol consumption, education level, self-assessed socioeconomic status, income level, marital status, and marital satisfaction.

### 2.4. Plan of Analysis

IBM SPSS Statistics 25.0 (IBM, Armonk, NY, USA) was used to analyze the data. First, our method for processing missing data was as follows. For demographic variables, we deleted individual cases with missing information. For the CES-D, we deleted cases with more than 10% missing data. For cases with 10% missing data or less, we used linear interpolation in SPSS to replace the missing data. Then, descriptive statistics of the depression scores and demographic characteristics of the Tibetans of the Qinghai–Tibet Plateau were calculated, and the overall characteristics of the sample and the distribution characteristics of the subjects according to different demographic variables were determined. Afterward, to investigate the gender differences in depression scores and prevalence rates among participants of different ages, we conducted a *t*-test and a chi-squared test for male and female participants of different ages.

The prevalence rate of depression was calculated by the following formula: the estimated detection rate of depression = (number of participants with depression)/n, where depression is defined by the CES-D scale score, as mentioned above, and n is the total number of completed questionnaires [48]. The result represents the estimated prevalence of depression in a population of adults on the Qinghai–Tibet Plateau. We focused on participants with depression according to the established cut-off and determined the prevalence rate of depression for each demographic variable. A chi-squared test was used to determine whether there were significant differences in the depression prevalence at the different levels of each demographic variable.

Finally, to investigate whether the various demographic characteristics were risk factors for depression, we used depression status as the dependent variable and demographic characteristics as the independent variable to conduct binary logistic regression for the whole sample. Because our data were cross-sectional, we report the prevalence rates as 95% confidence interval (CI) [49].

## 3. Results

### 3.1. Basic Information on the Depression Score and Demographic Characteristics

The range of the depression score was 0–25, the mean score was 8.49, the median score was 8.00, and the standard deviation was 6.91. The skewness coefficient was 0.27, and the kurtosis coefficient was −1.14. In this study, participants with depression symptoms (score ≥8) accounted for 52.3% of the sample, participants with depression (score ≥14) accounted for 28.6% of the sample, and participants with depression accounted for 54.7% of the participants with depressive symptoms. Other specific information about the sample is shown in Table 1.

### 3.2. Depression Scores and Prevalence among Participants of Different Genders and Ages

The depression scores and detection rate were higher for females than for males. The *t*-test and chi-squared test were used to verify the differences in depression scores and prevalence for different demographic variables. The chi-squared test results showed that there were significant gender differences in depression prevalence for the following age groups: 26 to 35 years, χ^2^ (1, *N* = 11,540) = 28.29; 36 to 45 years, χ^2^ (1, *N* = 6519) = 27.13; and 76 to 85 years, χ^2^ (1, *N* = 59) = 5.93. The *t*-test results showed that the gender differences in the depression scores of participants in all age groups except the 66- to 75-year and 86- to 94-year age groups were significant at the level of 0.05 or 0.01. One-way ANOVA showed significant differences in scores among all age groups (F = 69.88, *p* < 0.01). Multiple comparisons further revealed statistically significant differences among depression scores for all age groups except for the 76- to 85-year age group, whose depression scores did not differ significantly from those of the other groups. The prevalence of depression was highest in the 18- to 25-year age group and lowest in the 56- to 65-year age group. Further specific information is shown in Table 2.

### 3.3. Prevalence of Depression and Related Demographic Factors

Depression scores of 14 or above were considered indicative of depression (*N* = 6899), and the prevalence of depression with respect to different demographic variables is shown in Table 3. Depression was significantly correlated with gender, age, alcohol consumption, income level, self-assessed socioeconomic status, education level, marital status, and marital satisfaction (*p* < 0.01). To examine whether there were differences in depression prevalence at all levels of the different demographic variables, we used the chi-squared test. The results showed that the prevalence of depression was significantly different at different levels of different demographic variables. For the sake of concision, the remainder of the concrete results are presented in Table 3.

The prevalence of depression was found to be significantly different according to different demographic variables. To further explore the risk of depression associated with different demographic variables, we conducted a binary logistic regression analysis on the whole sample, and the results showed that although most demographic variables could predict depression symptoms, gender and marital satisfaction could not. Specifically, compared to non-drinkers, those who drank alcohol were more likely to be depressed (prevalence odds ratio (OR) = 1.57, 95% confidence interval (CI) [1.22, 2.02]; *p* < 0.01). Compared with participants with medium and high self-assessed socioeconomic status, participants with low self-assessed socioeconomic status were less prone to depression (prevalence OR = 0.14, 95% CI: [0.10, 0.18]; *p* < 0.01). Compared with illiterate participants and those with a secondary or high school education or above, participants who had only received a middle school education were more prone to depression (prevalence OR = 1.71, 95% CI: [1.49, 1.97]; *p* < 0.01). Participants with lower income levels were less likely to be depressed than those with higher and middle income levels (prevalence OR = 0.09, 95% CI: [0.04, 0.17]; *p* < 0.01). Unmarried participants were more likely to be depressed (prevalence OR = 4.11, 95% CI: [2.75, 6.16]; *p* < 0.01).

## 4. Discussion

This study adopted the short version of the self-reported CES-D to conduct the first large-scale survey of depression levels and risk factors among adult Tibetans in Qinghai Province, Yushu Tibetan Autonomous Prefecture. The high prevalence of depression among Tibetans living on the Qinghai–Tibet Plateau is likely due to cultural differences and chronic hypoxia due to the high altitude. Alcohol consumption, middle age, high self-assessed socioeconomic status, a middle school education level, high income, and being single may be risk factors for depression among Tibetans of the Tibetan Plateau.

### 4.1. Prevalence of Depression in Tibetan Adults

The prevalence of depression among Tibetans of the Qinghai–Tibet Plateau was 28.6% in our study. The result was higher than that reported in previous studies. Specifically, a summary of previous studies indicated that the prevalence of depression in Chinese people varies widely; for the general Chinese population, the prevalence of depression ranges from 5.3% to 23.8% [50,51,52]. A previous Western study showed that the prevalence of depression among high-altitude samples was 17.6% [53]. The depression prevalence in this study was higher than the Chinese norm and higher than that reported in a previous Western study of high-altitude sample. There are several possible reasons for this finding.

First, the severity of depression at high altitudes may be related to biological factors associated with hypoxia. Studies have shown that low pressure caused by high altitude is associated with mental state, fatigue levels, and neural activity [54,55]. Chronic hypoxia leads to changes in dopamine and serotonin in the monoamine system [56], and both dopamine and serotonin play a critical role in the pathophysiology and treatment of depression [57]. Moreover, recent research has suggested that mitochondrial dysfunction, such as mitochondrial respiration caused by hypoxia, may also play a role in depressive symptoms [58].

Second, the geographical environment associated with high altitude can also aggravate depression. A Chinese study of participants living at high altitudes in Qinghai Province showed that negative emotions increased as altitude increased, and obvious emotional disorders appear in the hypoxic environment present at high altitudes. These obvious symptoms of anxiety and depression are caused by the harsh natural environment on the plateau, with its cold and dry climate, hypoxic conditions, strong winds, and intense ultraviolet radiation, which make people more prone to negative emotions [59]. A previous study showed that the prevalence of anxiety and depression in participants living above altitudes of 4000 m was significantly higher than that of matched participants living at lower altitudes, indicating that high altitude causes the body to enter a long-term state of adverse stress [60]. In addition, the location is remote, and life is monotonous in the region. Both physical and mental deprivation had significant effects on the participants’ mental health. Therefore, the mechanism underlying the high prevalence of depression in the participants in this study may involve high altitude.

Third, previous studies have demonstrated that there are differences in the assessment of positive emotions in different cultural contexts when a self-rating scale is used. Asian culture emphasizes the suppression of positive emotions, and this difference affects the total scores on self-rated scales [61]. Positive emotion and negative emotion are not simply two poles of the subjective cognitive experience of emotion. The depressive symptoms caused by the absence of positive emotion and the aggravation of negative emotion are different in nature and quantity among participants from different cultural backgrounds [62,63,64]. The results of a factor structure analysis of a short version of the CES-D using Chinese participants showed that the CES-D scores of Chinese participants were indeed higher than those of participants in Western studies [42].

In addition, the high prevalence of depression in our research area may be related to the earthquake that occurred six years ago. In 2010, a 7.1-magnitude earthquake struck Yushu City in Yushu Prefecture. However, the April 14 Yushu earthquake spread to only two counties in Yushu Prefecture, and the sampling scope of this study comprised the entire prefecture. The area affected by the earthquake accounted for only 11.6% of the total area of Yushu Prefecture [65]. Although six years have passed since the earthquake, we cannot rule out the association between the high depression prevalence in this study and the influence of earthquakes in a small percentage of participants [66,67].

### 4.2. Depression in Tibetan Adults by Gender and Age

This study grouped the samples and specifically examined the differences in Tibetans’ depression levels by gender and age. Some of the findings deserve further comment. We observed that females had higher levels of depression than males, which is consistent with the results of many previous studies. Other studies using the CES-D [68,69,70], other depression scales [71,72,73], or related symptom scales [74,75,76] showed that females generally have higher levels of depression than males. Our findings are supported by an interview study focusing on independent highlands at the opposite edge of the Tibetan Plateau [77].

Additionally, females are more biologically susceptible to depression than males. According to the viewpoint of biological susceptibility, some specific chromosomes, such as 15q and 12 [78,79], and some genes [80,81], such as the serotonin receptor gene *HTR1A* [82], are associated with depression. In this study, females showed higher levels of depression than males, possibly because the theoretical path of gene–stress interaction was more significant in females than males [83]. In addition, depression scores may be partly related to differences in personality traits between males and females [84]. While females are expected to exhibit feminine gender characteristics by being delicate and emotional, males are culturally expected to be strong and independent and are encouraged to be more independent and autonomous [85]. When encountering negative life events, men tend to analyze them rationally and face them calmly and bravely, which affects their sensitivity to negative life events and negative emotions. However, females are generally more emotional than males, are more likely to become excited or be impulsive, and to have large emotional ups and downs and poor adaptability, thus generating more negative emotions and high levels of depression [86]. These results suggest that more attention should be paid to individual physiological gender identity in the prediction of depression and the development of interventions for adults on the Tibetan Plateau in the future.

Moreover, the female participants in this study may have had a higher prevalence of depression than male participants because of the different impacts of some depression-related diseases. For example, peripheral vascular diseases, such as lower extremity arterial disease, are often related to depression, especially in females [87].

Depression levels were also significantly different in different age groups. The 18- to 25-year-old subgroup had the highest depression level of all the subgroups, which may be related to the psychological characteristics of this subgroup. First, Angold [88] noted that, compared with older adults, younger adults report more emotional symptoms. Early adulthood is a critical period of transition from immaturity to maturity. Physiological and psychological changes, social role changes, and employment pressure can all become stressors, leading to psychological crises and increased depression levels. After this period, psychophysiological maturity tends to be reached, awareness of society and self is improved, psychological stress buffers and endurance capacity are enhanced, negative emotions are reduced, and depression levels also decrease [89]. Between the 36- to 45-year-old subgroup and the 66- to 75-year-old subgroup, there was a significant decline in the depression prevalence, which may be partly due to the change in social roles. As a normal part of getting older, the participants gradually changed from a state in which they were the primary support and pillar of their family to a state of idle retirement. With no work pressure, grown children who can support themselves, and reduced life pressures, these individuals experienced fewer negative emotions and had fewer depression symptoms [90]. However, the higher level of depression in those older than 76 years may be related to the physical quality of life of this subgroup. Previous studies have noted that biological factors play an important role in predicting depression in the elderly [91]. The underlying risk factors for depression change with age. Older people’s physical health may decline with age, leading to depression [92].

### 4.3. Demographic Differences in Depression among Tibetan Adults

In addition to gender and age, other demographic factors affecting depression were discovered. The illiterate participants had the lowest depression scores on average and were largely less depressed than the educated participants, partly because the participants in this study lived in a rural area characterized by a nomadic culture. In rural China, some people believe that “schooling is useless” due to limitations in terms of education level, knowledge and insight; such beliefs place educated participants under greater societal pressure, which results in their higher average depression scores [93]. Their higher depression levels may also result because highly educated people have a broader knowledge and perhaps higher expectations in various domains, and these unattainable expectations induce negative emotions, thus leading to higher depression scores on average [94]. Moreover, results in this study also indicated that the depression prevalence of high-income people was significantly higher than that of low-income people. This may be because, with the rapid development of China’s economy, high-income people (compared to low-income people) may face more stress events due to a fast-paced life. Compared with unmarried people, participants who were married to their original partners had the lowest average depression scores, possibly because they have a stable and relatively harmonious family environment. Due to environmental characteristics and clan factors, social networks in rural areas are usually based on kinship and related to religious beliefs [95]. On this basis, a good family environment, whether it provides internal interpersonal interaction or strong social support, may play an important role in inhibiting the development of depression [96].

### 4.4. Limitations

The study had several limitations. First, we used a self-evaluation questionnaire to measure the level of depression. Although it is common to measure depression with self-evaluation questionnaires [97,98,99], self-evaluation can lead to inaccurate measurement, which may have contributed to the high prevalence of depression in our study. Because our survey was a preliminary screening of depressive symptoms in the region, it cannot be used for clinical diagnosis. In future studies, a mixed methods approach, such as depression measures in conjunction with interviews, should be used. Second, factors that may impact the prevalence of depression, such as physical health (e.g., medical history, self-reported health status), oxyhemoglobin saturation, and the differences between urban and rural residents, were not measured [100,101]. In addition, to test the effect of high altitude, it would be better to compare the prevalence of depression in these samples with that of Tibetan participants living at lower altitudes. The lack of a matched group may also have made the results somewhat inaccurate. Moreover, there is a lack of research on the mental health of the people of Asia, especially those in East Asian minority areas, and most of the existing research is cross-sectional, with a lack of in-depth and long-term longitudinal follow-up research. Therefore, it is necessary to strengthen the research on the mental health of people in minority areas, especially in terms of longitudinal research. For the special group of ethnic minorities in plateau areas, we should pay attention not only to their mental health but also to the dynamic process of mental health changes. Notably, more effective interventions should be implemented to address the negative aspects of mental health in these populations and promote a good mental health status. These factors must be considered in future research.

## 5. Conclusions

This study is the first to recruit a large-scale sample of Tibetans living in high-altitude areas of the Qinghai–Tibet Plateau. The depression level among adults living on the Tibetan Plateau was investigated, and the possible risk factors were analyzed. The high altitude at which the participants live may have a negative impact on their depression levels. Marital status, income, education level, and self-assessed socioeconomic status all have a significant impact on depression level. In conclusion, this study on the depression status of Tibetans on the Qinghai–Tibet Plateau and the possible risk factors may help provide a new insight for designing prevention and intervention strategies. It may also be of great significance to conduct research on depression in other related groups.

## Figures and Tables

**Table 1:** Demographic information.

Characteristic	Number	M ± SD	Effective Percentage
Gender			
Male	9965	41.3	41.3
Female	14,176	58.7	58.7
Alcohol use (missing 670)			
Yes	22,944	95.0	95.0
No	527	2.2	2.2
Self-assessed socioeconomic status (missing 845)			
Low	523	2.2	2.2
Medium	15,792	65.4	65.4
High	6981	28.9	28.9
Educational level (missing 281)			
Illiterate	10,820	44.8	44.8
Junior high school or below	11,604	48.1	48.1
Polytechnic school or high school or above	1717	7.1	7.1
Income level (missing 281)			
Low	3748	15.5	15.5
Medium	20,058	83.1	83.1
High	54	0.2	0.2
Marital status			
Unmarried	1818	7.5	7.5
First marriage	21,543	89.2	89.2
Other	780	3.2	3.2
Marriage satisfaction (missing 870)			
Dissatisfied	407	1.7	1.7
Neutral	7	0.0	0.0
Satisfied	22,857	94.7	94.7

Note. Because of missing data for some of the demographic variables, the sum of the effective percentage is not equal to 100% in some cases.

**Table 2:** Depression scores and prevalence among participants of different genders and ages.

Age Group, Years	Whole Sample			Male			Female			*t*	η_p_^2^	χ^2^
	Number	Score	%	Number	Score	%	Number	Score	%			
18–25	3624	10.13 ± 5.44	32.5	1330	9.55 ± 5.79	32.0	2294	10.47 ± 5.19	32.8	−4.94 **	<0.01	0.22
26–35	11,540	7.61 ± 7.08	26.4	4949	6.83 ± 6.97	23.8	6591	8.19 ± 7.11	28.3	−10.27 *	0.01	28.29 **
36–45	6519	9.25 ± 7.32	34.1	2609	8.22 ± 7.30	30.4	3910	9.94 ± 7.25	36.6	−9.36 *	0.01	27.13 **
46–55	1798	8.42 ± 6.62	20.5	809	7.80 ± 6.84	21.0	989	8.92 ± 6.39	20.1	−4.59 **	0.01	0.22
56–65	369	7.82 ± 5.12	10.6	149	7.90 ± 5.70	13.4	220	7.77 ± 4.71	8.6	0.23 **	<0.01	2.15
66–75	214	7.46 ± 4.78	12.6	85	6.82 ± 5.02	14.1	129	7.88 ± 4.60	11.6	−1.59	0.01	0.29
76–85	59	9.05 ± 6.45	28.8	27	11.22 ± 7.11	44.4	32	7.22 ± 5.28	15.6	2.48 *	0.10	5.93 *
86–94	18	6.89 ± 5.94	22.2	7	8.71 ± 4.79	28.6	11	5.73 ± 6.51	18.2	1.04	0.06	0.27
Total	24,141	8.49 ± 6.91	28.6	9965	7.67 ± 6.94	26.2	14,176	9.08 ± 6.84	30.2	−15.74 **	0.01	45.76 **

Note. * *p* < 0.05. ** *p* < 0.01.

**Table 3:** Prevalence of depression and related demographic factors.

Characteristic	CES-D Score ≥ 14					
	Number	Effective Percentage	Prevalence	χ^2^	OR	95% CI
Gender				45.76 **		
Male	2614	37.9	26.2%		1.03	[0.96, 1.11]
Female	4285	62.1	30.3%		1.00	ref
Alcohol use				5.72 **		
Yes	6770	98.1	29.3%		1.57	[1.22, 2.02]
No	129	1.9	24.5%		1.00	ref
Self-assessed socioeconomic status (missing 114)				2856.11 **		
Low	98	1.4	18.7%		0.14	[0.10, 0.18]
Medium	2956	43.6	18.7%		0.21	[0.19, 0.22]
High	3731	55.0	53.4%		1.00	ref
Educational level				3085.38 **		
Illiterate	1201	17.4	11.1%		0.23	[0.20, 0.26]
Junior high school or below	5178	75.1	44.6%		1.71	[1.49, 1.97]
Polytechnic school or high school or above	520	7.5	30.3%		1.00	ref
Income level (missing 89)				523.76 **		
Low	514	7.5	13.7%		0.09	[0.04, 0.17]
Medium	6257	91.9	31.2%		0.18	[0.09, 0.34]
High	39	0.6	72.2%		1.00	ref
Marital status				43.70 **		
Unmarried	564	8.2	31.0%		4.11	[2.75, 6.16]
First marriage	6190	89.7	28.7%		2.13	[1.46, 3.13]
Other	145	2.1	18.6%		1.00	ref
Marriage satisfaction (missing 246)				22.70 **		
Dissatisfied	74	1.1	18.2%		0.70	[0.43, 1.12]
Neutral	1	0.0	14.3%		0.61	[0.07, 5.58]
Satisfied	6578	98.9	28.8%		1.00	ref

Note. * *p* < 0.05. ** *p* < 0.01. Because of missing data for some of the demographic variables, the sum of the effective percentage is not equal to 100% in some cases. CES-D = Center for Epidemiologic Studies Depression Scale; OR = odds ratio; CI = confidence interval; Ref = reference group.
